# The Role of Inflammasomes in LPS and Gram-Negative Bacterial Sepsis

**DOI:** 10.3390/jcm14197102

**Published:** 2025-10-09

**Authors:** Eduardo Hernández-Cuellar, Kohsuke Tsuchiya, Oscar Medina-Contreras, Ricardo Valle-Ríos

**Affiliations:** 1 Laboratorio de Biología Celular y Tisular, Departamento de Morfología, Universidad Autónoma de Aguascalientes, Aguascalientes 20100, CP, Mexico; 2 Division of Immunology and Molecular Biology, Cancer Research Institute, Kanazawa University, Kakuma-machi, Kanazawa 920-1192, Japan; ktsuchiya@staff.kanazawa-u.ac.jp; 3 Epidemiology, Endocrinology & Nutrition Research Unit, Hospital Infantil de México Federico Gómez, Mexico City 06720, CP, Mexico; omedina@himfg.edu.mx; 4 Research Division, Faculty of Medicine, Universidad Nacional Autónoma de México (UNAM), Mexico City 04360, CP, Mexico; vallerios@unam.mx; 5 Laboratory of Translational Research in Cellular and Molecular Biology, Hospital Infantil de México Federico Gómez, Mexico City 06720, CP, Mexico

**Keywords:** sepsis, LPS, Gram-negative bacteria, inflammasomes, DAMPs, caspase-11, GsdmD

## Abstract

**Background/Objectives**: Sepsis is a life-threatening condition characterized by an uncontrolled immune response due to systemic infections. It is responsible for millions of deaths worldwide. Although inflammasomes play an important role in host defense, they have a detrimental role in sepsis induced by LPS or Gram-negative bacteria. We aimed to revise the molecular mechanisms of inflammasome activation in sepsis by LPS and Gram-negative bacteria other than cytokine release as treatments blocking TNF-α and IL-1 cytokines have been ineffective even though cytokine storm is associated with lethality. **Results**: Studies with knockout mice deficient in inflammasome-derived cytokines have shown contrasting results on the role of these proinflammatory cytokines in the lethality of LPS- and Gram-negative-induced sepsis. However, DAMPs released after non-canonical inflammasome activation such as extracellular DNA, histones, HMGB1, and tissue factor result in disseminated-intravascular coagulation (DIC) and mortality in mice. Blocking these products in preclinical studies with animal models showed improved clinical scores and survival after LPS-induced sepsis or polymicrobial sepsis induced by Cecal Ligation and Puncture. **Conclusions**: Even though immunomodulatory drugs have shown inconclusive results as therapies for sepsis, blocking DAMPs associated with DIC may be considered for clinical trials in the future, especially in patients presenting biomarkers of coagulopathies.

## 1. Introduction

Sepsis is defined as a life-threatening condition induced by an uncontrolled immune response to infections leading to organ dysfunction [1]. According to the World Health Organization (WHO) from data reported in 2020, there were 48.9 million cases of sepsis and 11 million sepsis-related deaths worldwide, representing around 20% of all deaths [2,3]. From these deaths, more than 6 million were estimated to be due to bloodstream infections, respiratory tract infections, or intra-abdominal infections. Among the most relevant pathogens were *Staphylococcus aureus*, *Escherichia coli*, *Klebsiella pneumoniae*, *Streptococcus pneumoniae*, and *Pseudomonas aeruginosa* [4,5]. During sepsis, inflammatory responses are elicited by pathogen-associated molecular patterns (PAMPs) and danger-associated molecular patterns (DAMPs), which are recognized by receptors of the innate immune cells such as Toll-like receptors and nucleotide-binding oligomerization domain (NOD)-like receptors [6]. It generates cytokine release resulting in proliferation of leucocytes and adhesion to endothelial cells because of an augmented expression of adhesion molecules and chemokines. This alteration leads to endothelial dysfunction that together with the activation of coagulation cascades results in poor tissue perfusion, hypotension, and septic shock, a condition occurring in severe sepsis characterized by multi-organ failure and high mortality rates [5,7]. Interestingly, after the initial inflammatory response an immunosuppression state prevails. Indeed, postmortem studies of intensive care unit patients that died from sepsis showed decreased levels of CD4+ and CD8+ T cells. This immunosuppression renders patients susceptible to secondary infections and is associated with an early death [8,9]. Even though the cytokine storm has been mainly associated with lethality in bacterial sepsis, there is controversial data from the use of mice deficient in the expression of certain inflammatory cytokines and therapies blocking cytokines such as IL-1β and TNF-α have been ineffective in reducing mortality in patients. Hereafter, we will discuss the role of inflammasomes in host defense and in the severity of sepsis by LPS and Gram-negative bacteria with emphasis on those molecular mechanisms independent of cytokines leading to lethality.

## 2. Inflammasomes in Host Defense

**Figure 1:**
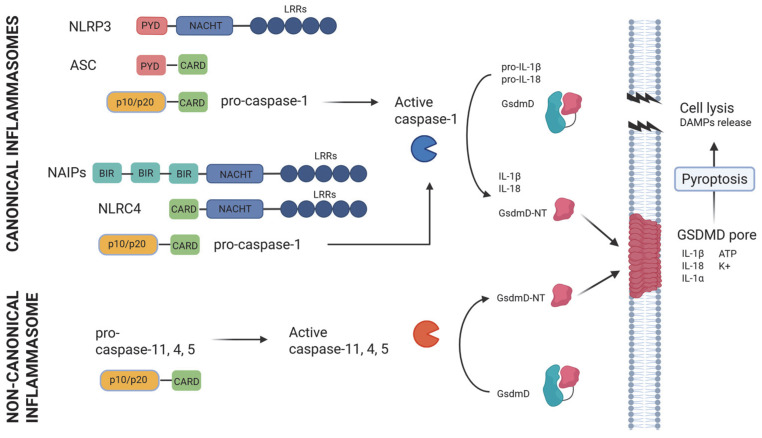
Inflammasomes activated by PAMPs from Gram-negative bacteria. Canonical inflammasomes Nlrc4 [14,15,16,17] and Nlrp3 [18,19,20] activate caspase-1 which processes pro-IL-18, pro-IL-1β, and GsdmD [12,13]. Caspase-11, -4, and -5 participate in the non-canonical inflammasome processing GsdmD [21,22,23]. The release of the GsdmD-NT fragment induces pore formation and pyroptosis.

Pattern recognition receptors are sensors of PAMPs and DAMPs including C-type lectin receptors (CLRs) and Toll-like receptors (TLRs), which are membrane-bound receptors, and cytosolic receptors such as retinoic acid-inducible gene I (RIG-I), absent in melanoma 2 (AIM2)-like receptors (ALRs), and NOD-like receptors [10]. Inflammasomes are intracellular multiprotein complexes serving as pattern recognition PRRs able to sense PAMPs or DAMPs [11]. NOD-like receptors (NLRs) are a family of inflammasomes containing a pyrin domain (PYD) or a caspase recruitment domain (CARD). These domains recruit directly or indirectly procaspase-1. For example, NLRs containing a CARD domain bind procaspase-1 directly, while NLRs containing a PYD domain bind procaspase-1 through an apoptosis-associated speck-like protein containing CARD (ASC), which contains both CARD and PYD domains [6]. Activation of caspase-1 is necessary for processing and releasing IL-18 and IL-1β. Also, caspase-1 activates GsdmD, releasing its N-terminal fragment, responsible for the induction of membrane pores and an inflammatory cell death called pyroptosis (Figure 1) [12,13].

Some Gram-negative bacteria such as *Salmonella typhimurium*, *Pseudomonas aeruginosa*, *Shigella flexneri*, and *Legionella pneumophila* activate the Nlrc4 (NLR family CARD domain-containing protein 4) inflammasome [14]. Previously, Nlrc4 inflammasome was described as a sensor of the bacterial flagellin and the type III secretion system protein PrgJ [15]. Indeed, NAIP proteins act as sensors of these bacterial products before recruiting Nlrc4, ASC, and pro-caspase-1, forming a multiprotein complex. For example, NAIP5 and NAIP6 interact with Nlrc4 to sense flagellin, while PrgJ is sensed by NAIP2-Nlrc4 [16,17]. The Nlrc4 inflammasome is critical for host defense against enteric bacteria such as *Salmonella*. Also, Nlrc4 is expressed in intestinal macrophages and dendritic cells reacting against pathogenic bacteria but maintaining tolerance with commensal bacteria which fail to activate this inflammasome (tolerance). In contrast, the activation of the Nlrc4 inflammasome by pathogens induces the secretion of IL-1β, which in turn enhances the expression of adhesion molecules in the intestinal endothelium recruiting neutrophils to combat the infection [14,24]. On the other hand, gain-of-function mutations in the *nlrc4* gene are associated with autoinflammation with infantile enterocolitis due to an overactivation of this inflammasome. Patients present gastrointestinal symptoms with recurrent autoinflammation and febrile episodes [25].

Similarly, the Nlrp3 inflammasome is activated by PAMPs from viral, bacterial, and fungal origin. Also, it is activated by endogenous and exogenous DAMPs. Examples of endogenous DAMPs include ATP, monosodium urate, amyloid-β, cholesterol crystals, oxidized mitochondrial DNA, etc. Alum and silica are examples of exogenous DAMPs [18]. Due to the different nature of these DAMPs and PAMPs, it is not possible that Nlrp3 acts as a direct sensor of these unrelated molecules; instead, the Nlrp3 inflammasome is somehow activated by some cellular alterations including lysosomal leakage, mitochondrial dysfunction, reactive oxygen species (ROS) production, and changes in ion fluxes (i.e., K^+^ efflux). For this reason, Nlrp3 is considered a non-classical PRR [19]. As a result, this inflammasome is very versatile, participating in host defense against microbes, non-infectious inflammatory conditions, and autoimmune diseases originated by gain-of-function mutations of the *nlrp3* gene [20].

Other types of inflammasomes include AIM2 and NLRP1 inflammasomes. The Nlrp1 inflammasome belongs to the NOD-like receptor family and was the first inflammasome identified. In mice, Nlrp1b acts as a sensor of the anthrax lethal toxin from *Bacillus anthracis*. In humans, Nlrp1 participates in host defense against some viral infections and is associated with autoimmune and inflammatory diseases due to polymorphisms of the *nlrp1* gene. The AIM2 inflammasome senses cytosolic dsDNA and participates in host defense against viruses and bacteria. Pyrin, a receptor encoded by the *mefv* gene, forms a non-classic inflammasome as it does not sense PRR directly; instead, it senses inactivation of Rho GTPases by bacterial toxins [26]. The participation of these inflammasomes in sepsis by LPS and Gram-negative bacteria is less clear and documented and is not discussed in this review.

Furthermore, inflammatory caspases such as mouse Caspase-11 and its human homologs Caspase-4 and-5 participate in the non-canonical inflammasome acting as intracellular sensors of lipopolysaccharide (LPS) [27]. The expression of caspase-5 and -11 is low and induced by LPS in contrast to the relatively constitutive expression of caspase-4 [28]. The optimal ligand for caspase-11 and caspase-4/5 is hexa-acylated lipid A from LPS, but caspase-4 can also sense tetraacylated lipid A. Some bacteria such as *Yersinia* and *Francisella* present under-acylated lipid A to evade TLR recognition. This means that caspase-4 recognition of less acylated lipid A is a host defense mechanism combating the evasion strategies by these bacteria [29]. Caspase-4 and -5 but not caspase-11 were shown to process pro-IL1β and pro-IL-18 in Gram-negative bacterial infections [30]. This suggests differences between rodents and humans in terms of non-canonical inflammasome activation that need further investigation.

After binding LPS, these caspases activate themselves and cleave GsdmD, releasing the N-terminal fragment that promotes pyroptosis [21]. The resulting K^+^ efflux is sufficient to activate the NLRP3 inflammasome with the consequent caspase-1 activation and the processing of pro-IL-1β and pro-IL-18. Intracellular Gram-negative bacteria such as *E. coli*, *C. rodentium*, and *Vibrio cholera* activate the non-canonical inflammasome [22]. Caspase-11 is required for innate immunity against cytosolic bacteria but not vacuolar bacteria. For example, the *Salmonella typhimurium* sifA mutant and *Legionella pneumophila* sdhA mutant failed to stay in the vacuoles, escaping to the cytosol and activating caspase-11, resulting in bacterial clearance in a way independent of Nlrp3, Asc, and Nlrc4. This mechanism works for *Burkhoderia* species that invade cytosol naturally [23].

**Figure 2:**
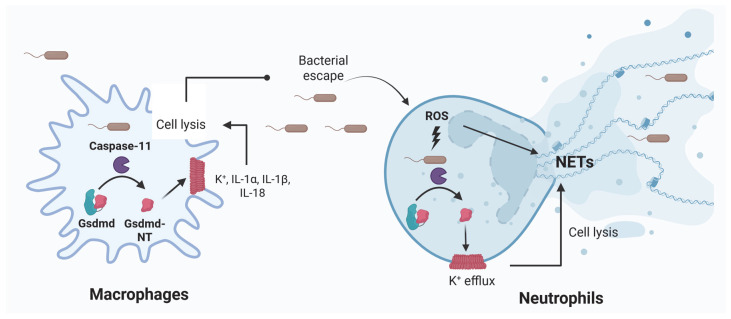
Non-canonical inflammasome in host defense against Gram-negative bacteria. After LPS recognition by caspase-11, the processing of GsdmD induces pore formation and pyroptosis in macrophages, allowing the escape of intracellular bacteria, eliminating their replication niches and exposing them to the action of neutrophils which in turn kill bacteria through ROS and NETs. Bacterial dissemination is also blocked by NETs [31,33].

In addition, caspase-11 activation has protective roles independent of cytokine release. In this regard, infection with *Salmonella* ΔsifA induces cell death and NET extrusion in neutrophils after non-canonical inflammasome activation. Interestingly, treatment with deoxyribonuclease I dissolved the NETs increasing the bacterial burden in WT-mice but not in Caspase-11-/- and GsdmD-/- mice, showing the relevance in host defense of the GsdmD-dependent extracellular trap release [31]. Also, GsdmD activation has direct effects on bacteria as gasdermin-N domain promotes binding and pore-forming activity on lipid membranes including those found in bacteria such as cardiolipin and phosphatidylethanolamine [32]. Furthermore, pyroptosis was the mechanism responsible for bacterial clearance in infection models with intracellular bacteria [33,34], and this clearance was independent of the secreted IL-1β and IL-18. Instead, cell death allowed the release of bacteria from macrophages with the subsequent uptake and bacterial killing by neutrophils through reactive oxygen species (Figure 2) [33]. Altogether, inflammasomes confer protection against bacterial infections by enhancing immune responses, eliminating replication niches, and direct bacterial killing; however, in the next sections we will review their detrimental roles in sepsis.

## 3. Inflammasomes in Sepsis Induced by LPS

LPS is the main component of the outer membrane in Gram-negative bacteria. It consists of lipid A, a hydrophobic domain, followed by a polysaccharide hydrophilic core, and a distal oligosaccharide called O-Antigen. The natural tolerance of mouse strain C3H/HeJ to lethal doses of LPS was due to mutations in the TLR4 receptor (TLR4) [35,36]. Lipid A binds to the lipopolysaccharide binding protein (LBP) and to the cell surface protein CD14, and this complex is recognized by immune cells through TLR4 together with the myeloid differentiation factor 2 (MD-2). This interaction induces the activation of the MyD88 and TRIF signaling pathways resulting in the expression of inflammatory cytokines, chemokines, and type I interferons [37]. Thus, the TLR4 signaling pathway is critical in the inflammatory response that occurs in LPS-induced sepsis.

In addition, caspase-1 was initially shown to be responsible for the maturation of IL-1β and secretion of IL-1β and IL-1α, both in vitro in macrophages treated with LPS, and in vivo in an endotoxic shock model in mice stimulated with high doses of LPS. Also, caspase-1 ko mice were resistant to endotoxic shock [38]. Similarly, treatment with VX-166, a broad caspase inhibitor, showed inhibition of the release of IL-1β and IL-18, and mice had an improved survival with a lethal dose of LPS and with Cecal Ligation and Puncture (CLP), a procedure to induce polymicrobial sepsis [39]. Furthermore, mice deficient in ASC or NLRP3 were more resistant to sepsis induced by an intraperitoneal injection with LPS in comparison with wild-type mice [40,41]. Thus, not only TLR4 signaling but also the Nlrp3/caspase-1/IL-1 axis were described as important for the immune response and lethality of LPS-induced sepsis. Indeed, these pathways are somehow connected as the expression of Nlrp3 and pro-IL-1β is induced with LPS through TLR4 signaling, a step necessary before the activation of the Nlrp3 inflammasome [18,19].

Later, in a major finding, it was shown that caspase-11 was responsible for the activation of caspase-1 and IL-1β secretion in macrophages infected with intracellular Gram-negative bacteria or LPS [22]. Furthermore, mice lacking caspase-11 were protected from a lethal dose of LPS independent of caspase-1 [22,42]. This was different from the finding in which caspase-1 loss was responsible for the protection against LPS-induced sepsis; it was explained that some caspase-1-deficient mice came from strain 129, which harbors a mutation in the caspase-11 locus, giving similar results to caspase-11-deficient mice [22]. Thus, the real reason for the susceptibility of mice to LPS-induced sepsis is the presence of caspase-11 rather than caspase-1. Interestingly, in an endotoxemia model consisting in priming with LPS or poly(I:C) and a secondary challenge with LPS (sublethal dose), only caspase-11-deficient mice were resistant, while WT- and TLR4-deficient mice succumbed [43,44]. Therefore, cytoplasmic contamination with LPS is responsible for the activation of caspase-11 independent of TLR4, and the susceptibility seems to be due to the pro-caspase-11 expression induced by TLR signaling, the activation of the non-canonical pathway by LPS, and the subsequent inflammatory response.

It is known that after LPS binding, caspase-11 activation, and pore formation by GsdmD, inflammatory cytokines are released through these pores [21,45]. Also, changes in ion fluxes result in cell swelling, cell lysis, and the release of DAMPs. Interestingly, GsdmD-deficient mice were protected from a lethal dose of LPS [21]. Thus, the lethality induced by GsdmD in sepsis could be due to the released products such as IL-1 cytokines or DAMPs [46]. However, the role of IL-1 cytokines in the septic shock induced by LPS is controversial. It was shown that IL-1ra (IL-1R antagonist) reduced the lethality of LPS in rabbits in a dose-dependent manner [47]. On the other hand, IL-1RI-/- mice were as susceptible as wild-type mice to LPS [48]. Also, IL-1α-, IL-18-, and IL-1β-deficient mice were not resistant to the LPS-induced endotoxic shock [46,49], and IL-1β-/- mice were only resistant when a pre-treatment with a low-dose LPS was used before challenging with a high-dose LPS [50]. Thus, because of these discrepancies, the contribution of the IL-1 cytokines to lethality in sepsis must be further investigated.

Nevertheless, there must be other roles of inflammasomes associated with the lethality of LPS-induced sepsis independent of the cytokines. Indeed, the hepatocyte-released HMGB1 binds LPS and favors its internalization into macrophages and endothelial cells via RAGE (receptor for advanced glycation end-products), and deletion of the *hmgb1* gene in hepatocytes or neutralization of extracellular HMGB1 with antibodies improved the survival of mice in LPS-induced endotoxemia and bacterial sepsis [51]. HMGB1 provides another way of LPS delivery into the cytosol and caspase-11/4/5 activation besides that of LPS delivery after bacterial phagocytosis and phagosomal escape [6]. Furthermore, recent evidence suggests that released DAMPs including extracellular DNA and DNA-binding proteins such as histones and HMGB1 are associated with disseminated intravascular coagulation (DIC) [52]. Interestingly, extracellular histones contribute to lethality in sepsis by inducing endothelial dysfunction, hemorrhage, and thrombosis in mice challenged with LPS or in polymicrobial sepsis induced by CLP. The lethality was reverted with antibodies to histones or activated protein C (APC), an anticoagulant protein [53]. Furthermore, the cytoplasmic presence of LPS activates the non-canonical inflammasome in neutrophils, inducing GsdmD-dependent cell death and extrusion of NETs, which in turn induce platelet and erythrocyte aggregation, fibrin deposition, and thrombosis leading to the development of DIC [54,55]. Similar results were found in sepsis regardless of the bacterial type (Gram-positive or Gram-negative). A DNase infusion resulted in a better outcome with an improved microvascular perfusion [55]. It was also found that the non-canonical inflammasome activation by LPS generates DIC by increasing the activation of tissue factor (TF or coagulation factor III) via calcium influx and phosphatidylserine exposure. In this case, TF activation was GsdmD-dependent but independent of pyroptosis [56]. Phosphatidylserine (PS) normally appears in the cytoplasmic leaflet of cellular plasma membranes, but during cell death is located on the outer leaflet. This abnormal exposure of PS was found in leucocytes, erythrocytes, platelets, and endothelial cells of patients with sepsis, and was associated with TF activation and coagulation [57]. Interestingly, blocking TF with neutralizing antibodies resulted in protection against coagulation and lethality [58,59]. Therefore, released DAMPs may enhance cell death and inflammation, and the resulting DIC may certainly lead to organ failure and mortality.

## 4. Inflammasomes in Bacterial Sepsis

Since early in the 1990s the role of IL-1β in bacterial sepsis was analyzed, as high circulating levels of this cytokine were detected after an injection with live *E. coli* in healthy baboons. The administration of IL-1ra improved the hemodynamic changes and survival [60]. Later, Nlrp3-/- mice or WT mice injected with an Nlrp3 inhibitor were resistant to sepsis induced by *E. coli* challenge [61]. Interestingly, caspase-1-/- mice but not IL-1β-/- or double IL-1β/IL-18-deficient mice were protected against an intraperitoneal challenge with live *E. coli* [62]. It is now known that inflammasome activation is detrimental in bacterial sepsis, but as in LPS-induced sepsis, the results related to the role of the inflammasome-derived cytokines in the severity of sepsis are still debatable.

A polycaspase inhibitor improved survival in mice after CLP. The blood count of several Gram-positive and Gram-negative bacterial species were highly diminished with caspase inhibition [63]. Mechanistically, it was mentioned that sepsis induced apoptosis of lymphocytes, and the survival of mice treated with the polycaspase inhibitor depended on lymphocytes as RAG-1-deficient mice presented very high amounts of bacteria in blood and showed higher mortality rates regardless of the treatment; also, transfer of T lymphocytes expressing Bcl-2 improved survival [63]. In addition, with the CLP model, mice deficient in Nlrp3 showed improved survival and had lower bacterial burden in several tissues. Neutrophil depletion with anti-Ly-6G antibody blocked the protection of the Nlrp3-deficient mice, indicating the role of neutrophils in bacterial clearance when the inflammatory response coming from this inflammasome is suppressed. It was mentioned that neutrophils from Nlrp3-/- mice were not affected in number but showed augmented phagocytosis [61]. Thus, in bacterial sepsis the resulting inflammation is associated with apoptosis of lymphocytes, deficient neutrophil function, reduced bacterial clearance, and mortality.

The role of the Nlrc4 inflammasome in sepsis is poorly understood even though it is highly expressed in the peripheral blood of patients with this condition [64]. In a sepsis-like model in mice with dysbiosis in which a multidrug-resistant *E. coli* pathobiont spread systemically, the NAIP5/NLRC4 inflammasome was responsible for lethality. The inflammatory response rather than bacterial loads in the gut was suggested as the cause of lethality [65]. Also, silencing Nlrc4 in vivo with siRNA reduced lung injury and inflammation in a septic shock model in mice using CLP [66]. Therefore, it seems that the Nlrc4 inflammasome is detrimental in bacterial sepsis, but the literature related to this point is still scarce and needs further investigation.

As mentioned for LPS-induced sepsis, other mechanisms associated with inflammasome activation besides inflammatory cytokines may account for the severity of bacterial sepsis. For instance, baboons infected with *E. coli* showed an increase in circulating histone levels, renal failure, and death. Lethality was reverted with antibody to histones or activated protein C (APC) [53]. Also, the inner rod protein EprJ from *E. coli* (a type III secretion system protein) induced systemic blood clotting and massive thrombosis in mice due to the release of tissue factor (TF) during pyroptosis of macrophages in a way dependent on caspase-1 and GsdmD but independent of IL-1β and IL-18. Indeed, coagulation by *E. coli* infection was prevented in GsdmD-/- mice [58]. Blocking of TF with neutralizing antibodies protected against coagulation and lethality induced by *E. coli* or EprJ [58,67]. As mentioned, PS exposure on cells is associated with cell death and activation of TF; and milk fat globule-EGF factor 8 (MFG-E8), a molecule that binds PS, improved the survival rate of mice exposed to polymicrobial sepsis [68]. Altogether, besides inflammatory cytokines, the DAMPs released during cell death are associated with disseminated blood clotting accounting for the lethality of bacterial sepsis (Table 1).

## 5. Therapies with Immunomodulatory Effects in Sepsis

Sepsis treatment has made progress in the last decades due to a better understanding of the pathophysiology of this condition. However, severe sepsis still has poor prognosis, and management to keep the hemodynamic status (heart rate, blood pressure, arterial oxygen saturation, and respiratory rate) controlled is still challenging. Conventional treatment includes the use of antibiotics, fluid resuscitation with crystalloids, and vasopressors. Cytokine release is associated with endothelial dysfunction and coagulopathies that lead to hypotension, poor tissue perfusion, and organ failure. Thus, it is quite logical to consider blocking cytokines and other inflammatory molecules as treatments for sepsis. Meta-analyses studies using monoclonal antibodies to block TNF-alpha in patients with sepsis have shown inconclusive results regarding the benefit of this therapy to improve survival rates [69,70,71]. Similar results were obtained when trying to block IL-1 or platelet-activating factor (PAF) as treatments for sepsis [72,73,74]. Robey, R.C., et al. published a systematic review and meta-analyses about several immunomodulatory drugs used as treatment for sepsis, revising safety, adverse effects, and effectiveness in reducing mortality. In this study the immunomodulatory drugs belong to a different category such as anti-inflammatory drugs, complement inhibitors, cytokine inhibitors, immune cell stimulators, and platelet-activating factor antagonists. Results showed that anti-inflammatory drugs had the highest effect on reducing short-term mortality followed by cytokine inhibition while TLR4 antagonists were associated with adverse events [75]. New therapies with immunomodulatory effects include nangibotide, a peptide that inhibits TREM-1, a receptor found in some immune cells necessary for the activation of NF-κB. However, this therapy is still in phase II and needs further investigation because of variations in the results obtained [76]. Also, thymosin α1 is a peptide secreted by the thymus acting as an immunomodulator. It was shown a benefit in reducing short-term mortality with thymosin α1; however, results are inconclusive because of sample sizes and heterogeneity in results among groups [77]. These results highlight the complexity of sepsis and its treatment. The heterogeneity in results may be due to different populations analyzed without considering specific biomarkers in patients that could be targets for more personalized treatments.

## 6. Conclusions

Inflammasomes play a key role in host defense against bacterial infections; however, we have reviewed their detrimental role in sepsis induced by Gram-negative bacteria or their product LPS. It seems to be that cytokines released during inflammasome activation are not the main cause of lethality. Instead, the released DAMPs such as extracellular DNA, histones, HMGB1, NETs, and TF induce disseminated intravascular coagulation leading to multiorgan failure and death. The literature mentioned that sepsis management has progressed significantly over the past years but is still considered a life-threatening condition with poor prognosis, and some therapeutic approaches are still debated. Blocking experimentally these compounds in preclinical studies with animal models has resulted in a better outcome after LPS or bacterial challenge. Even though immunomodulatory therapies have not had the expected results in reducing mortality, blocking the release or the action of these DAMPs may be considered in the management of sepsis in the future, especially as a complement to the conventional therapy, and in patients showing biomarkers of coagulopathies.

## Figures and Tables

**Table 1:** Inflammasome-derived DAMPs involved in the lethality of sepsis.

Inflammasome-Derived DAMPs	Action in LPS and Bacterial Sepsis Leading to Lethality
Hepatocyte-derived HMGB1	Promotes internalization of LPS into macrophages and endothelial cells via RAGE receptor, activation of caspase-11, and lethality in mice. Neutralizing HMGB1 or *hmgb1*-deficient mice were protected from polymicrobial sepsis and endotoxemia [51]. HMGB1 induces DIC [52].
Histones	Induce endothelial dysfunction, hemorrhage, and thrombosis in LPS and polymicrobial sepsis. Lethality was reduced in mice by neutralizing antibodies to H4 or activated protein C (APC) [52,53].
NETs	LPS and bacteria induce NETs extrusion in neutrophils. NETs cause platelet- and erythrocyte-aggregation, fibrin deposition, and thrombosis leading to DIC in mice. Removal of NETs with a DNase infusion coagulation and improved tissue perfusion [52,54,55].
Tissue Factor (TF)	LPS induces non-canonical inflammasome activation and phosphatidylserine (PS) exposure, resulting in TF activation and DIC. Neutralization of TF and PS prevents LPS-induced DIC and reduces mortality in mice [56,57,58,59].

## Abbreviations

The following abbreviations are used in this manuscript:

DAMPs	Damage-associated molecular patterns
PAMPs	Pathogen-associated molecular patterns
LPS	Lipopolysaccharide
NETs	Neutrophil extracellular traps
GsdmD	Gasdermin D
HMGB1	High Mobility Group Box 1
